# ArqPy: A Python Toolbox for Remote Sensing Image Preprocessing and AI-Assisted Interpretation of Derived Products for Archaeological Prospection

**DOI:** 10.3390/s26175478

**Published:** 2026-08-29

**Authors:** Cristian Iranzo, Paula Uribe, Jorge Angás, Fernando Pérez-Cabello

**Affiliations:** 1 Departamento de Geografía y Ordenación del Territorio, Facultad de Filosofía y Letras, Universidad de Zaragoza, 50009 Zaragoza, Spain; fcabello@unizar.es; 2 Instituto Universitario de Investigación en Ciencias Ambientales de Aragón (IUCA), Universidad de Zaragoza, 50009 Zaragoza, Spain; 3 Departamento de Ciencias de la Antigüedad, Facultad de Filosofía y Letras, Universidad de Zaragoza, 50009 Zaragoza, Spain; uribe@unizar.es (P.U.); j.angas@unizar.es (J.A.); 4 Instituto de Patrimonio y Humanidades (IPH), Universidad de Zaragoza, 50009 Zaragoza, Spain; 5 Fundación Agencia Aragonesa para la Investigación y el Desarrollo (ARAID), 50018 Zaragoza, Spain

**Keywords:** crop marks, multispectral imagery, very high-resolution satellite imagery, image preprocessing

## Abstract

**Highlights:**

**What are the main findings?**

- ArqPy uses an object-oriented architecture that generates suitable derived products from WorldView-3 imagery for initial archaeological assessment while facilitating the future integration of additional satellite sensors.
- ArqPy integrates AI-based tools for complementary tasks, including MAE feature analysis of derived products, Z-PNN pansharpening, and SAM 3 text-guided segmentation of candidate crop marks.

**What are the implications of the main findings?**

- The workflow improves reproducibility and comparability in archaeological remote sensing by making preprocessing and enhancement steps explicit and easily deployable on standard Windows systems.
- ArqPy generated complementary WorldView-3-derived products that supported expert identification of candidate crop marks, while SAM 3 provided text-guided segmentation for preliminary detection of crop-mark-like features.

**Abstract:**

Remote sensing is widely used in archaeology, but the lack of standardised and readily deployable preprocessing workflows limits reproducibility and cross-study comparability, particularly for very high-resolution multispectral imagery. This study presents ArqPy, a Python toolbox designed to automate and standardise image preprocessing, enhancement and initial interpretation for archaeological prospection. The toolbox includes atmospheric correction, conventional pansharpening, spectral indices, principal component analysis (PCA), and spatial filtering. Its object-oriented architecture currently supports WorldView-3 (WV3) and WorldView Legion (LEGION) imagery and facilitates the future integration of additional sensors. ArqPy also incorporates complementary AI-based tools: Masked Autoencoder (MAE) feature analysis for exploring and ranking derived products, Z-PNN for deep-learning-based pansharpening, and SAM 3 for text-guided segmentation of candidate crop marks. The toolbox was applied to the preliminary inspection of crop marks at the Zar Tepe archaeological site in southern Uzbekistan before the 2026 field campaign. This case study illustrates the proposed workflow and demonstrates how ArqPy provides a reproducible and readily deployable environment through which archaeologists can access advanced image-processing methods. AI-based tools can support preliminary reconnaissance, but they cannot replace expert interpretation without further training and validation using site-specific archaeological data.

## 1. Introduction

Remote sensing has become a core method in archaeology because it provides rapid, non-destructive observation of the ground surface across large areas and multiple spatial scales. It supports research that ranges from mapping individual sites to studying regional settlement systems and landscape change. It also supports heritage monitoring, including erosion, urban expansion, and conflict-related damage, using repeated satellite acquisitions and long image archives. These roles are widely reported in reviews of archaeological and cultural-heritage remote sensing [1,2,3,4,5,6].

However, raw satellite images are rarely suitable for direct archaeological interpretation. Radiometric differences between acquisitions and sensors complicate comparisons across time [7,8,9]. Atmospheric conditions, illumination, seasonality, and vegetation phenology can mask subtle traces, such as crop or soil marks [10,11]. In complex landscapes, rich spectral information can also increase confusion, because non-archaeological factors may produce similar spectral responses [7,11]. These limitations motivate systematic correction and enhancement steps before interpretation and classification.

This need has increased with the spread of very high-resolution multispectral satellites. Sensors such as WV3 provide fine spatial detail and additional spectral bands, including SWIR, that can improve sensitivity to vegetation stress, soil moisture, and material differences relevant to buried features [10]. At the same time, these data often require careful radiometric handling, robust atmospheric correction, and pansharpening choices that balance spatial detail against spectral distortion [12]. Multi-temporal use further increases the need for consistent preprocessing [13,14].

Current practice shows partial convergence in the types of methods used, but limited standardisation in how they are implemented and reported [15]. Simple image-based atmospheric corrections are common for index-based studies, while pansharpening choices vary. Comparative studies inside and outside archaeology indicate that no single method is optimal across sensors, landscapes, and research objectives [16,17,18]. In addition, terms such as “spectral enhancement” are used inconsistently, which reduces cross-study comparability unless the transformations and parameters are explicitly reported [19,20]. These issues support the case for transparent, reproducible workflows and shared best practices [21,22,23].

A further constraint is practical deployment. Many archaeological teams work with limited computational support and heterogeneous software environments [22,24]. Lightweight tools that run on standard operating systems like Windows can reduce barriers to adoption and make workflows more consistent across users. This is aligned with broader calls for explicit, shareable pipelines and improved documentation of processing steps and parameters in archaeological digital practice [25,26,27,28,29].

Besides preprocessing and the generation of accurately corrected remote-sensing products, several approaches have aimed to identify areas likely to host archaeological remains through the detection of crop marks using automated or semi-automated methods [30,31]. Crop marks arise from differential vegetation growth caused by buried remains that affect soil moisture and nutrient availability, and they have traditionally been identified through expert visual interpretation of aerial photographs [32,33]. Vegetation indices such as NDVI and red-edge-based metrics are widely used to enhance stress patterns [34,35,36], while PCA and related transforms are applied to separate crop, soil, and background components in heterogeneous landscapes [30,37,38]. More recent approaches integrate image segmentation, geometric feature extraction, and learning-based models to reduce subjectivity and improve scalability [27,31], although detection performance remains strongly dependent on crop phenology, environmental conditions, and expert validation [14,35]. This progression motivates pipelines that can generate and rank multiple derived channels, enabling probabilistic identification of areas with high crop mark density rather than reliance on single indices or visual inspection alone.

This paper presents ArqPy, a toolbox that standardises key preprocessing operations for archaeological remote sensing. Its application is illustrated through a case study at the archaeological site of Zar Tepe, Uzbekistan, where it was used to identify crop marks in preparation for forthcoming field campaigns. The toolbox performs atmospheric correction and pansharpening for WV3 and LEGION imagery and generates derived products, including vegetation indices, PCA products, and spatially filtered images. It also integrates complementary AI-based methods, including MAE feature analysis, Z-PNN deep learning pansharpening, and SAM 3 text-guided segmentation of candidate crop marks. Its object-oriented design facilitates the integration of additional sensors while reducing the need to write new code or adopt different processing platforms. A total of 244 derived products were visually examined, resulting in the identification of four previously undocumented candidate crop marks and the selection of the products most informative for their interpretation at Zar Tepe.

## 2. Materials and Methods

### 2.1. Study Area

**Figure 1:**
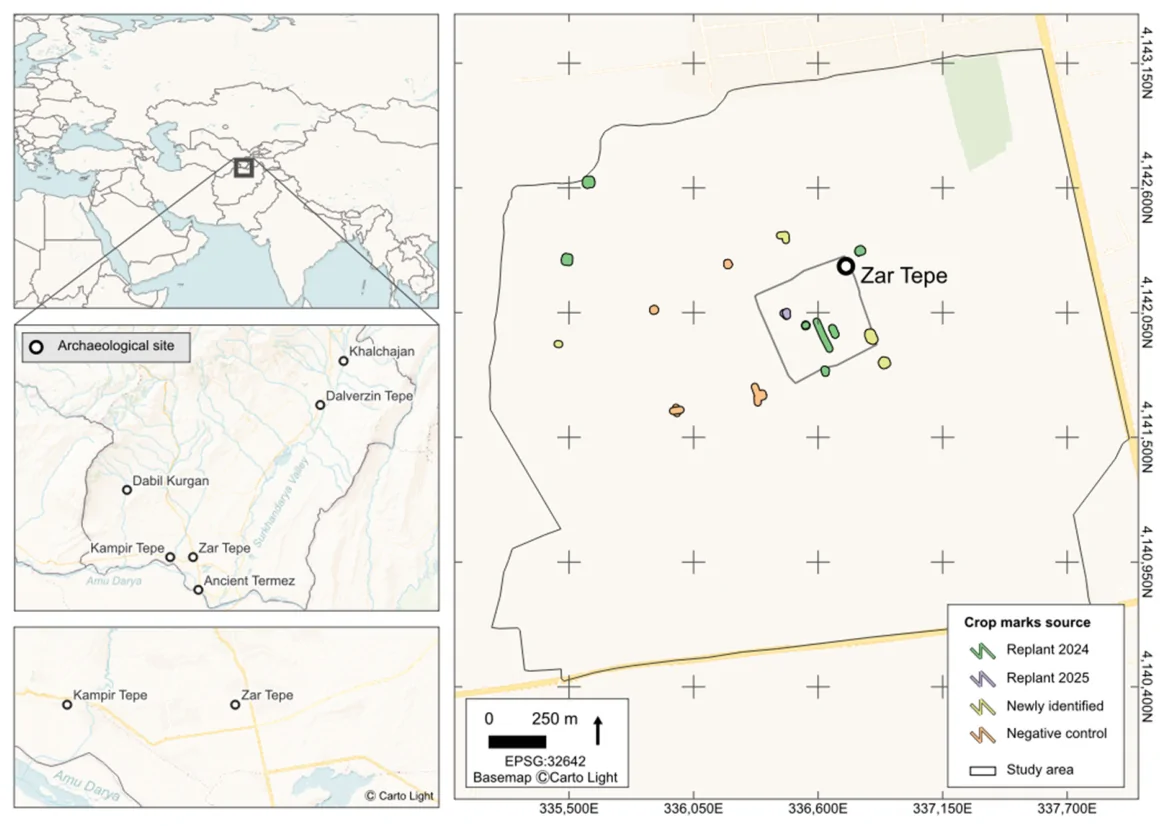
Location of the Zar Tepe archaeological site and study area. The mapped features include crop marks verified during fieldwork, four candidate crop marks newly identified and digitised using ArqPy-derived products, and four crop-mark-like negative controls selected to assess false-positive responses in the MAE saliency maps.

The toolbox was applied at the archaeological site of Zar Tepe, dated to the Kushan and Kushan-Sasanian periods (1st–5th centuries AD). The site is located in the Surkhandarya Province of southern Uzbekistan, within the northwestern sector of ancient Bactria (Figure 1). The Kushan period in Bactria (1st–3rd centuries AD) has been widely described as a phase of political stability and economic prosperity, reflected in the growth of valley settlements and the architectural complexity of urban centres [39,40]. From the mid-3rd century AD, the region progressively entered the Sasanian sphere of influence, followed by the arrival of Hunnic groups during the 4th–5th centuries AD [41].

Zar Tepe is a fortified settlement, roughly square in plan (~400 m per side; ~16.9 ha), enclosed by a defensive wall and an external ditch. The study area encompasses the settlement and its surroundings, covering a total area of 619.8 ha. It lies about 36 km northeast of Termez and close to other major Kushan-period sites, including Kampyr Tepe and Dalverzin Tepe. The surrounding landscape is part of a wide irrigated plain north of the Hindu Kush, supplied by the Amu Darya (ancient Oxus River) and its tributaries, particularly the Surkhan Darya. This region contains a high density of archaeological sites, with a notable concentration dating to the Kushan period. For a visual characterisation of the study area, including ground-level photographs from previous campaigns, see Angás et al. [42].

### 2.2. Toolbox Overview

A Python-based processing pipeline was developed to preprocess very high-resolution satellite imagery and to generate a set of derivative products. The workflow supports imagery acquired by WV3 and LEGION, and integrates tools from Orfeo ToolBox (OTB) [43] and GDAL [44], within a unified and automated framework. The toolbox is openly distributed through the Zenodo repository, together with documentation, source code, and configuration files [45].

The pipeline outputs bottom-of-atmosphere (BOA) reflectance imagery and multiple derivative datasets, including pansharpened products, principal component analysis (PCA) images, spectral indices, spatially filtered layers and saliency maps derived from deep-learning-based analysis. ArqPy also provides graphical interfaces for two complementary AI-based tools. The Z-PNN module [46] performs deep-learning-based pansharpening, and SAM 3 [47] applies text-guided segmentation to identify candidate crop marks. The last module requires additional configuration, including dedicated Python environments, model dependencies and pretrained weights; some operations also benefit from GPU acceleration. Once configured, it can be executed through the toolbox interfaces. ArqPy outputs are intended to support archaeological interpretation, with specific focus on the detection and characterisation of crop marks.

The software architecture is modular and object-oriented. Computational routines implementing image processing operations are separated from sensor-specific metadata handlers. The design allows the extension of the toolbox to additional sensors by implementing new metadata classes while reusing existing processing functions.

**Figure 2:**
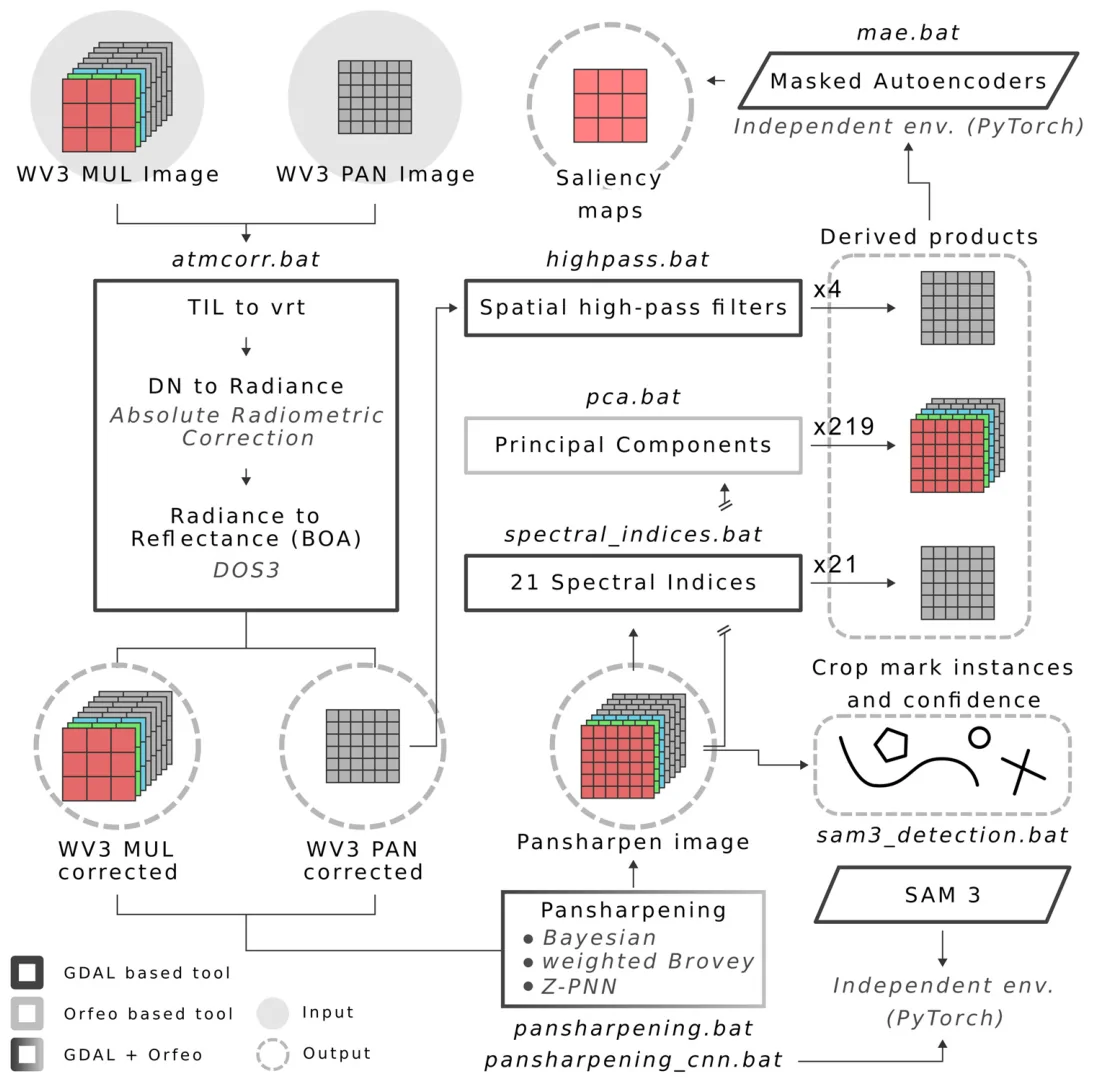
Toolbox architecture and relationships among processing components. The diagram represents the predefined workflow. First, the original very-high-resolution imagery, including multispectral and panchromatic bands, is radiometrically corrected. The corrected panchromatic band is used to generate four high-pass-filtered products. Pansharpening is then performed using one of the available conventional or AI-based methods. The resulting image can be processed with SAM 3 to segment candidate crop mark instances. Spectral indices and principal components are also computed from the pansharpened image to generate additional derived products. The derived products can subsequently be analysed using MAE-based saliency maps. Border colours indicate the software used by each operation, including GDALversion 3.12 and OrfeoToolbox version 9.1.1.

The toolbox is distributed as a set of standalone Windows executable files in BAT format, with each file corresponding to a specific task (Figure 2):

1. *atmcorr* for atmospheric correction;
2. *pansharpening* for spatial-spectral fusion;
3. *pansharpening_cnn* to perform deep-learning-based pansharpening using Z-PNN;
4. *pca* for principal component generation;
5. *spectral_indices* for index computation;
6. *highpass* for spatial filtering;
7. *mae* to generate Masked Autoencoder (MAE)-based saliency maps;
8. *sam3_detection* to apply SAM 3 text-guided segmentation.

**Figure 3:**
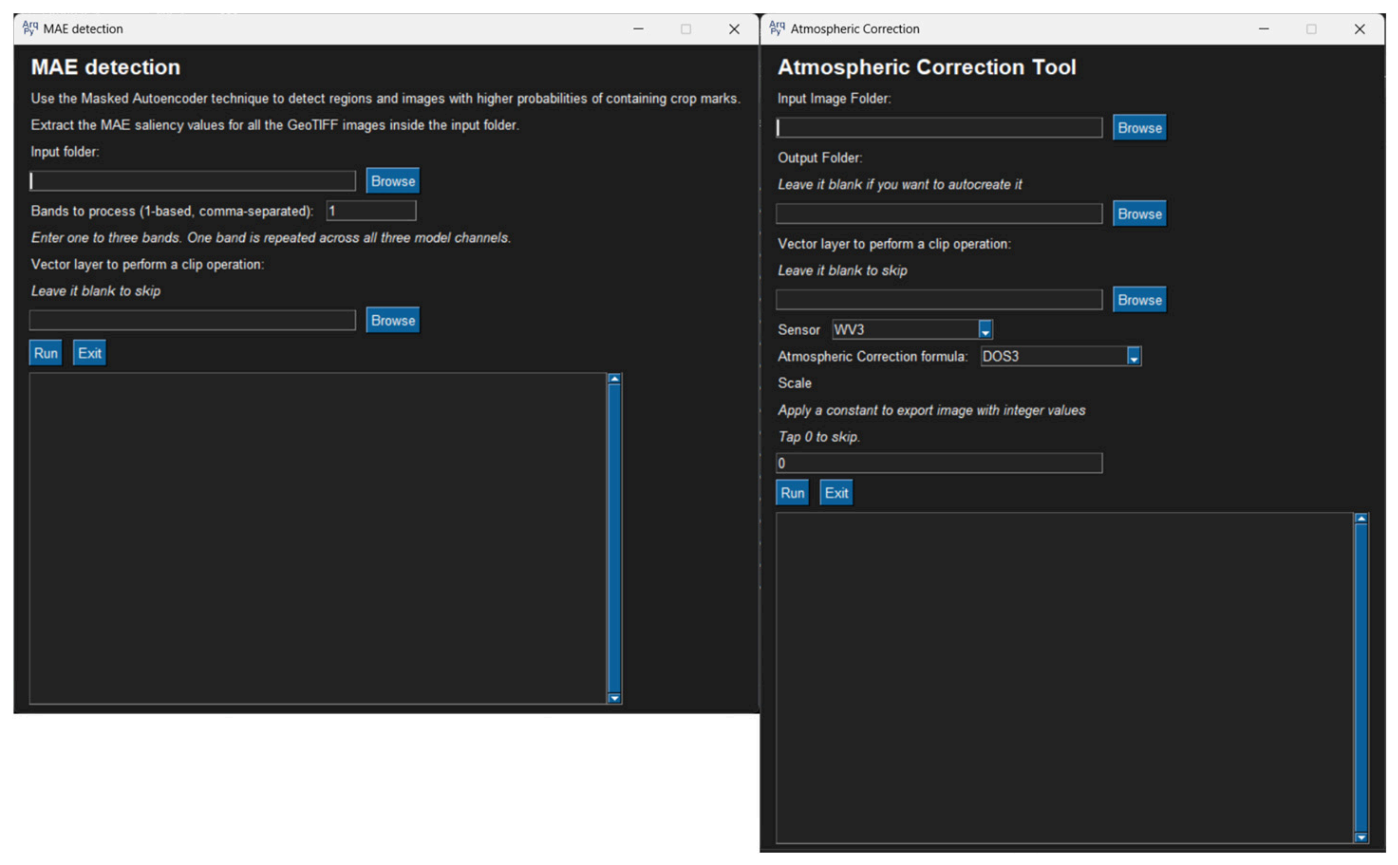
Examples of graphical user interfaces provided by ArqPy: the MAE saliency tool (*mae*), shown on the left, and the atmospheric correction tool (*atmcorr*), shown on the right. The interfaces provide input selection and operation-specific parameters, including source bands, an optional clipping layer, output settings, and the sensor from which the imagery was acquired.

Each executable includes a graphical user interface that allows non-expert users to define processing parameters (Figure 3). By specifying the directory containing raw imagery as provided by the data distributor, the full workflow can be executed without manual intervention. The MAE, SAM 3, and Z-PNN modules run in separate PyTorch version 2.13 environments.

All tools were developed and tested in isolated Python environments under Windows 11. The required libraries are packaged and distributed together with the executables. While the released binaries are Windows-specific, the toolbox can be ported to other operating systems by recreating the environments and installing the source code as described in the repository [45].

### 2.3. Image Preprocessing

Satellite imagery requires preprocessing to reduce radiometric, atmospheric, and geometric distortions and to ensure that pixel values are physically comparable across scenes. This step is essential in archaeological applications, where accurate spatial alignment and radiometric consistency are required for comparison with ancillary datasets, such as geophysical surveys [12].

The described application focuses on WV3 and LEGION sensors, whose images are distributed without a complete set of radiometric and atmospheric corrections. Additional preprocessing is therefore required prior to analysis. The presented pipeline implements a standardised and reproducible correction workflow applicable to both sensors, differing only in the sensor-specific radiometric metadata used.

**Figure 4:**
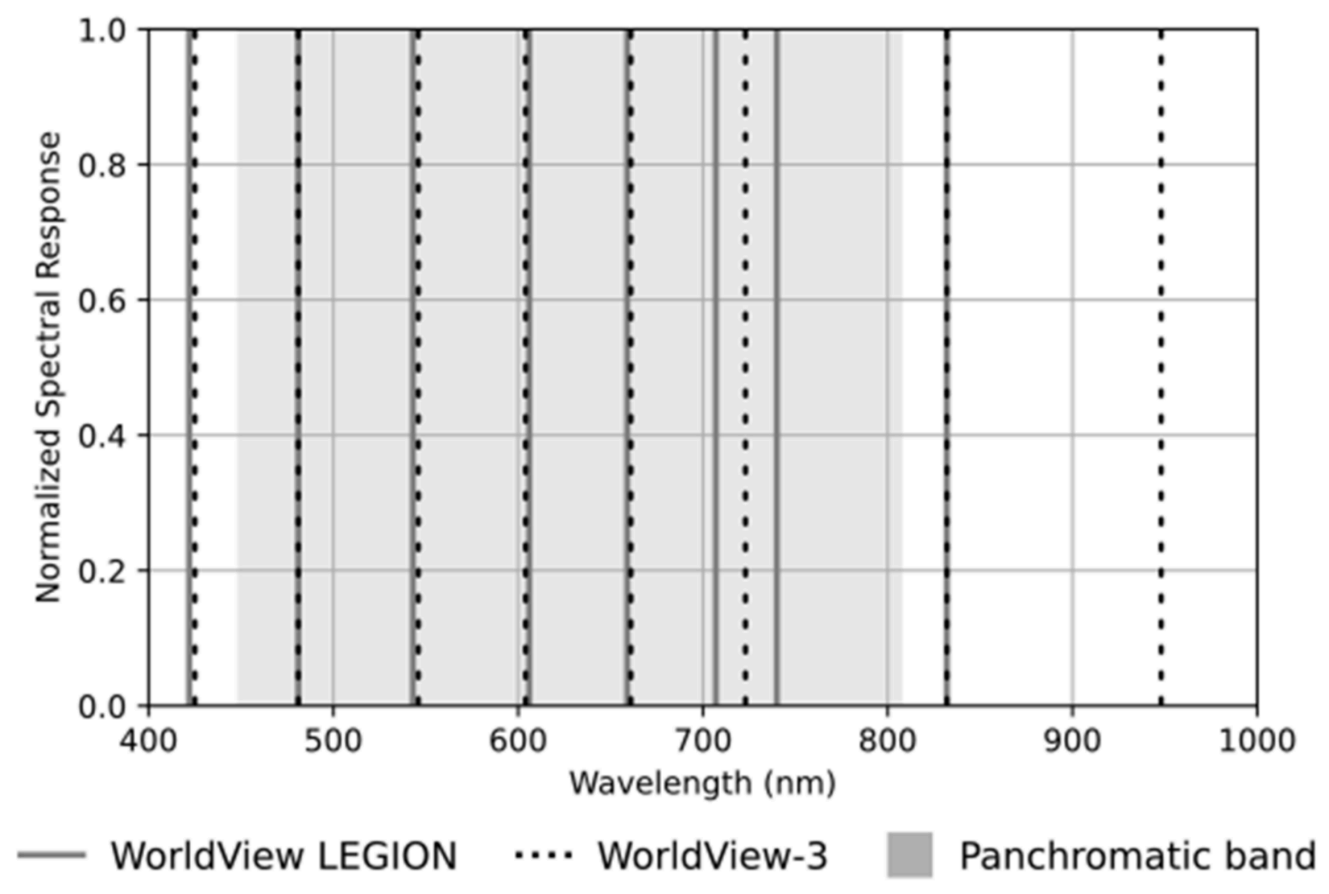
Spectral bands available in WorldView-3 and WorldView Legion satellites.

WV3 provides a panchromatic resolution of 0.31 m and a multispectral resolution of 1.24 m, whereas LEGION provides corresponding resolutions of 0.34 m and 1.36 m. Compared with WV3, LEGION includes an additional red-edge band and omits one of the near-infrared bands, resulting in a modified spectral configuration. The complete band specifications for both sensors are shown in Figure 4.

The first operation in this workflow is atmospheric correction. It was applied using the *atmcorr* executable and the Dark Object Subtraction method (DOS3 [48]). Digital numbers were first converted to top-of-atmosphere radiance and subsequently to bottom-of-atmosphere reflectance. Atmospheric path radiance was estimated from dark pixels using percentile-based thresholds, following an extension of the original method proposed by Chavez [49]. To reduce the risk of overcorrection caused by anomalously dark pixels or image noise, percentile-based dark-object values were used instead of absolute minimum values: the second percentile was selected for the multispectral bands, whereas the 0.1 percentile was applied to the panchromatic band. These percentile values were derived from digital numbers and converted to radiance prior to correction. Incident transmittance was computed as a function of band central wavelength and solar zenith angle, allowing wavelength-dependent correction [48].

After atmospheric correction, an optional step allows the atmospherically corrected images to be cropped to a region of interest. Whether cropped or not, the resulting images are then exported as Cloud Optimised GeoTIFFs using lossless compression.

Pansharpening was then performed using the *pansharpening* executable to enhance the spatial resolution of the multispectral imagery. Three methods are available. The first is a combination of a weighted Brovey method [50], applied to bands with high spectral correlation to the panchromatic signal, and a local mean and variance normalisation (LMVN) approach, applied to the NIR2 band, which shows lower correlation (Figure 4). These outputs were merged into a single multispectral product optimised for visual interpretation. The weighted Brovey method was implemented using GDAL, following coefficients proposed by Belfiore et al. [50].

The second pansharpening method available in the toolbox is a Bayesian approach. For analytical products, such as spectral indices and PCA, this approach is recommended because it better preserves spectral fidelity [51].

**Figure 5:**
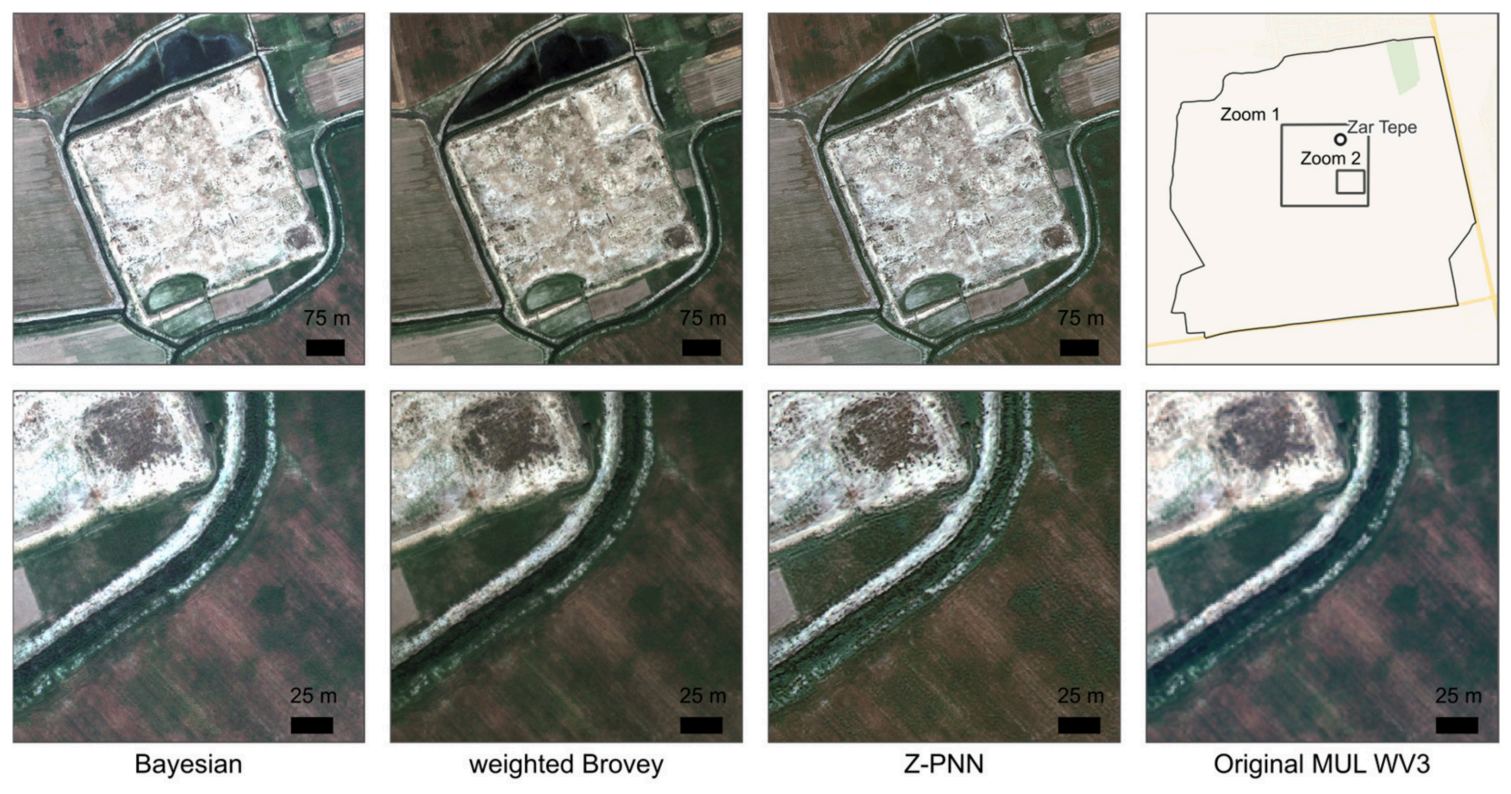
Comparison of pansharpening results for the original WV3 image acquired on 29 May 2017. The outputs are shown at two detail levels: Level 1 in the upper row and Level 2 in the lower row. Their locations are indicated in the overview map at the upper right, while the original image is shown at the lower right. All images are displayed using a linear contrast stretch of the mean ± three standard deviations (µ±3σ).

The third option is Z-PNN (Zoom-PNN), a CNN-based method that operates at full resolution and combines sensor-specific pretraining with rapid adaptation to the target image [46]. In tests on an off-training WV3 image, Z-PNN reached a spatial loss of approximately 0.06 in fewer than 200 iterations, whereas A-PNN-TA-FR required approximately 2000 iterations. Its initial spatial loss was already relatively low (approximately 0.10), although fine-tuning further improved spectral consistency. In this study, Z-PNN was applied using 10 target-adaptation iterations. A comparison of the pansharpening strategies is shown in Figure 5.

### 2.4. Generation of Derived Image Products

The derived image products generated with the toolbox are intended to enhance subtle spatial and spectral variations potentially associated with buried archaeological features. The corrected and pansharpened imagery serves as input for this stage.

Using the pansharpened image, the *spectral_indices* executable produces a total of 21 spectral indices. These indices were selected to enhance variations in crop stress and moisture conditions, which may reflect the presence of subsurface archaeological structures under suitable environmental conditions. All near-infrared bands were treated equivalently and assigned to the NIR1 band to ensure consistency in index computation. The full list of spectral indices and their corresponding formulations is available in the toolbox repository [45], in the directory *ArqPy-Toolbox*/*Toolbox*/*app*/*spectral_indices*/*data*/.

Principal component analysis (PCA) was performed using the *pca* executable, with the pansharpened image as input. The workflow uses the Orfeo ToolBox Dimensionality Reduction algorithm. All unique combinations of sensor spectral bands, with a minimum of three input bands, were evaluated, resulting in 219 band combinations for each available sensor. For each band combination, a corresponding set of principal components was generated.

Finally, the *highpass* tool generates a set of spatial high-pass-filtered outputs designed to enhance linear and textural features. In the predefined workflow, a corrected image is used as input, and four filters are applied: Laplacian, Laplacian of Gaussian (LoG), Sobel (horizontal), and high-boost filtering. These filters emphasise edges and fine-scale structures while suppressing low-frequency background variations. More generally, the toolbox allows the automated application of all available filter kernels to any GeoTIFF file, with user-defined selection of the spectral band to which the filters are applied.

### 2.5. Masked Autoencoder Analysis

**Figure 6:**
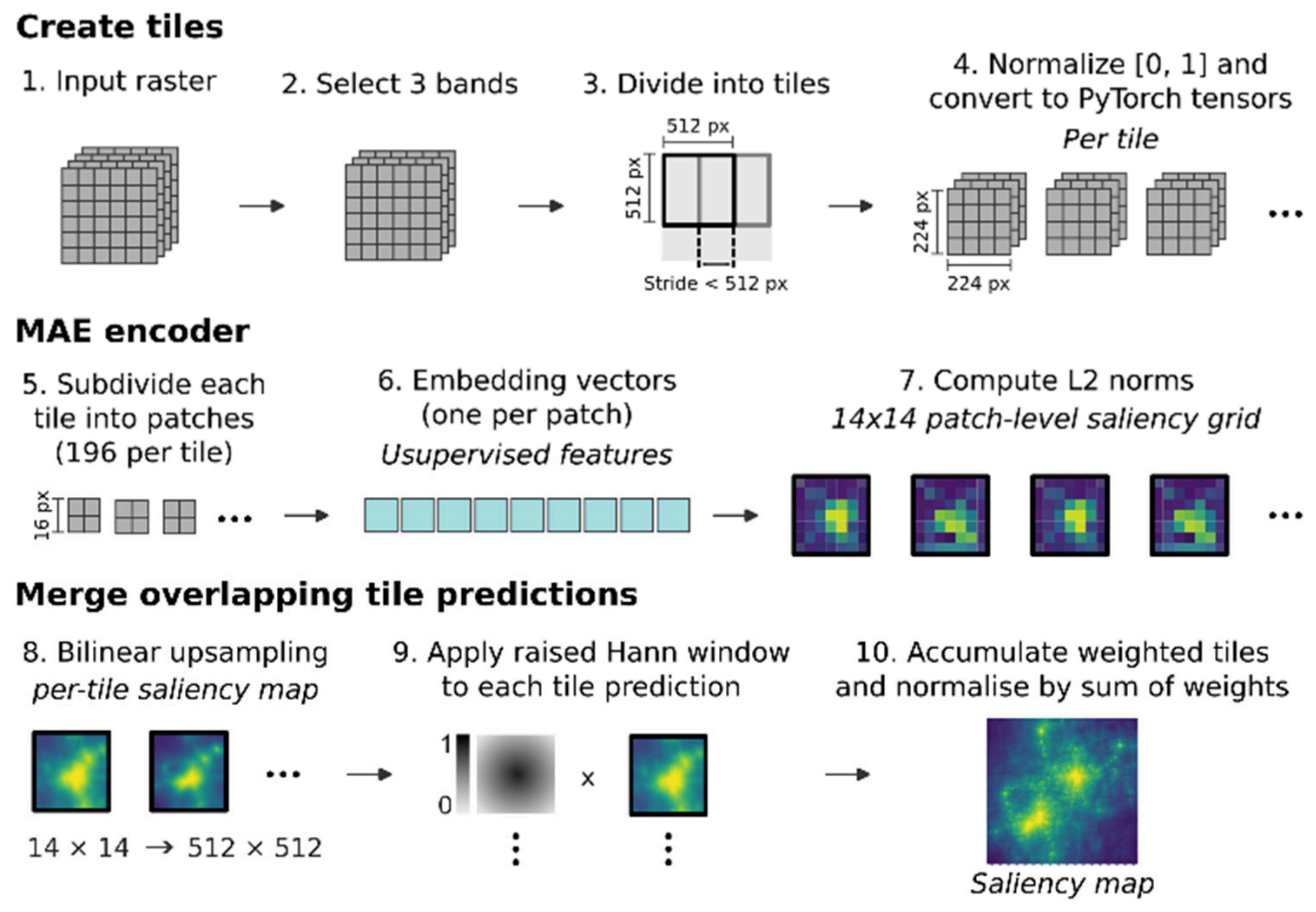
Workflow for MAE-based saliency map generation using overlapping tile-based inference. Each derived image product was divided into 512 × 512 pixel tiles with a stride smaller than the tile size, producing overlap between adjacent tiles. For each tile, patch-level saliency values were predicted on a 14 × 14 grid and bilinearly interpolated to the original tile resolution. Overlapping tile-level maps were then merged using a raised Hann weighting window to reduce border artefacts and generate a continuous saliency map for the full image.

A Masked Autoencoder (MAE) encoder [52] was used to identify image regions with a high density of geometric and textural features potentially associated with buried archaeological remains (Figure 6). A Vision Transformer-based MAE encoder [53], pretrained on ImageNet, was employed as a fixed feature extractor using the *mae* executable. No fine-tuning, masking, or image reconstruction was performed; the pretrained encoder was used only to extract patch-level feature representations. The executable allows the automated application of the MAE encoder to any GeoTIFF file.

Each derived image product was processed using an overlapping tile-based inference strategy. Input rasters were divided into 512 × 512 pixel tiles, with a stride smaller than the tile size to ensure overlap between adjacent tiles. Before being passed to the encoder, each tile was converted to a three-channel input when necessary, normalised independently by band to the [0, 1] range, and resized to the 224 × 224 pixel input size required by the MAE-pretrained Vision Transformer. The resized tile was subdivided by the model into 16 × 16 pixel patches, producing a 16 × 16 grid of 196 patch embeddings per tile. These embeddings encode high-level representations of local texture, contrast, and geometric structure learned during pretraining.

Patch-level feature saliency was quantified using the L2 norm of each embedding vector produced by the MAE encoder (Equation (1)). The L2 norm measures the magnitude of each patch embedding in feature space and was used as a proxy for local structural complexity. Higher norm values indicate patches with stronger geometric or textural content, whereas lower values correspond to more homogeneous or low-information areas. This metric provides a simple and computationally efficient measure of unsupervised saliency, without requiring inter-patch comparisons, labelled data, or additional model training.

(1) $${\mathrm{norm}}_{i}={\left\|e_{i}\right\|}_{2}=\sqrt{\sum_{j=1}^{n}e_{i,j}^{2}}$$

The resulting 14 × 14 patch-level saliency grid was bilinearly upsampled to the original 512 × 512 tile resolution. To obtain a continuous raster-scale saliency map and reduce artefacts at tile borders, overlapping tile predictions were merged using a raised Hann weighting window. This weighting assigns higher importance to the central region of each tile and lower importance to tile margins, where border effects are more likely to occur. For each raster pixel, the final MAE saliency value was computed as the weighted average of all overlapping tile predictions (Equation (2)). S(x,y) is the final saliency value at pixel location (x,y), $S_{i}(x,y)$ is the saliency value predicted from tile i, and $w_{i}\left(x,y\right)$ is the corresponding Hann-window weight. A small minimum weight was retained to ensure stable normalisation in areas covered by only one tile. The final output was exported as a single-band floating-point GeoTIFF.

(2) $$S\left(x,y\right)=\frac{\sum_{i}w_{i}(x,y)S_{i}(x,y)}{\sum_{i}w_{i}(x,y)}$$

From each MAE-derived saliency map, summary statistics including the maximum and mean saliency values were computed and stored together with the corresponding derived image product identifier. These MAE-based statistics constitute the primary quantitative criterion used to rank derived products and to guide the initial identification and interpretation of potential crop mark signatures.

### 2.6. SAM 3 Text-Guided Segmentation

SAM 3 [47] was integrated as an optional tool to support the preliminary identification of candidate crop marks and soil marks (Figure 2). The pansharpened RGB image was processed using overlapping 1008 × 1008-pixel tiles with an overlap of 128 pixels. Prior to inference, each tile was independently contrast-stretched between its 2nd and 98th percentile values. Four text prompts were evaluated: crop marks, soil marks, archaeological crop marks, and archaeological soil marks. For each prompt, predictions were generated using two confidence thresholds (0.50 and 0.70), and only instances with confidence scores equal to or greater than the specified threshold were retained.

Predictions from overlapping tiles were subsequently merged to produce a binary candidate mask and a confidence raster, while individual detections and their associated attributes were recorded in a CSV file. These outputs were considered model-generated candidate detections rather than definitive archaeological features and therefore required subsequent expert archaeological interpretation. The SAM 3 module was implemented in a separate PyTorch environment and requires access to the pretrained SAM 3 checkpoint. Detailed instructions for configuring and running the module are provided in the accompanying repository [45].

### 2.7. Validation

**Figure 7:**
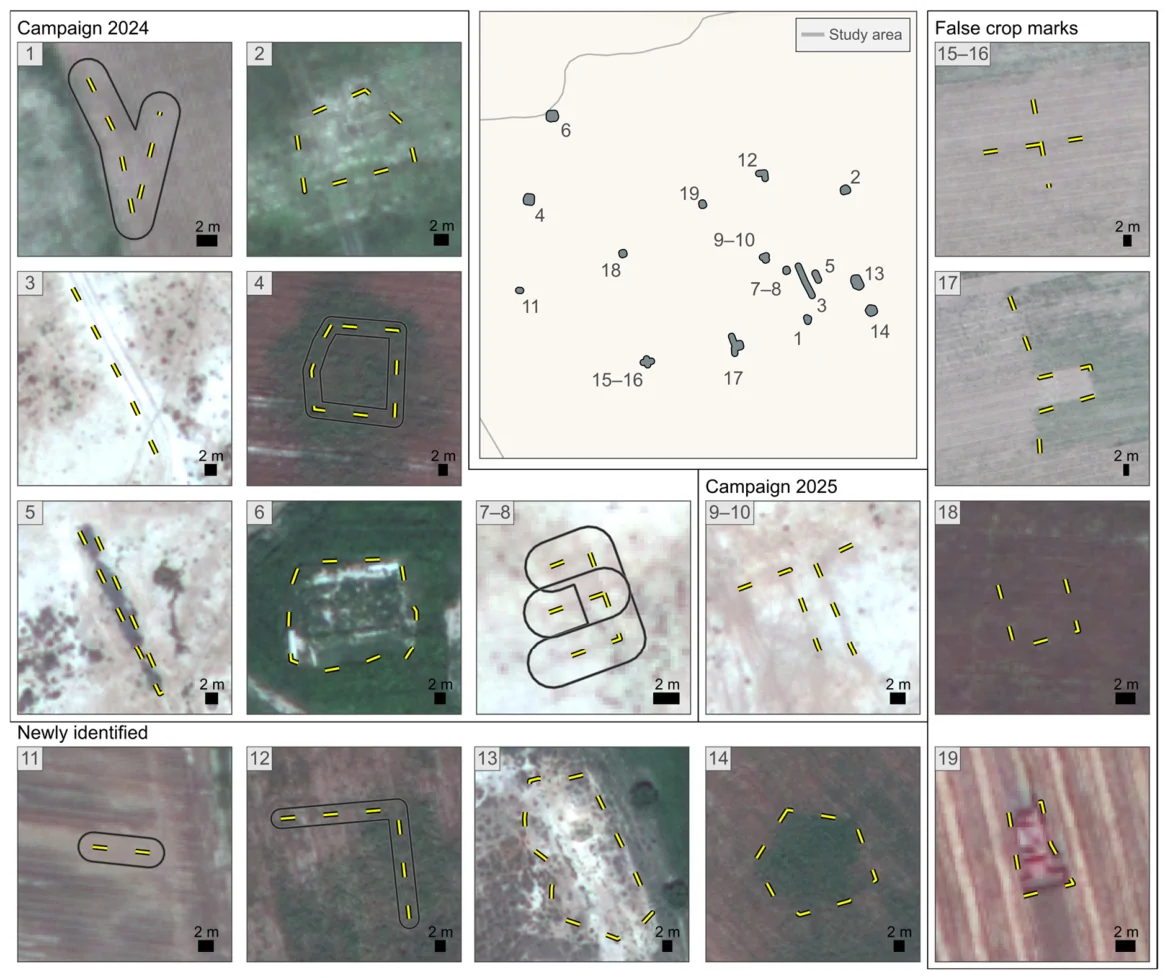
Locations of the crop marks and negative controls used in this study. Features are grouped according to the field campaign in which they were recorded, whether they were newly digitised from ArqPy-derived products, or whether they served as negative controls. The background is the natural-colour pansharpened WV3 image used for validation. Crop marks are outlined with yellow dashed lines; those outlined in black were not visible in the natural-colour composite and required derived products for their identification (IDs 1, 4, 7, 8, 11, and 12).

Validation was conducted to assess the responses of the MAE saliency maps and SAM 3 segmentation when applied to crop-mark identification. The reference dataset comprised 19 digitised features (Table 1): 10 field-documented crop marks (eight in 2024 and two in 2025) recorded using a GNSS device and a customizable mobile application configured for archaeological field recording [42,54], four candidate crop marks were identified through expert inspection of ArqPy-derived products, and five negative controls (Figure 7). Three negative controls (IDs 15, 16, and 18) were placed in areas without visible geometric features. The remaining controls represented non-archaeological geometric patterns: ID 17 followed a boundary within a harvested field, and ID 19 surrounded a tractor.

The validation was conducted using all derived products generated from a WV3 image acquired on 29 May 2017. The image was atmospherically corrected, cropped to the study area, and pansharpened using the Bayesian method, which provided the closest visual agreement with the visible spectrum of the original multispectral image (Figure 5). Processing was performed on a Windows 11 workstation equipped with an Intel^®^ Core™ i7-10700 CPU at 2.90 GHz, 16 GB of RAM, and 1 TB of local storage.

For each MAE saliency raster, all valid pixels intersected by each digitised feature were extracted, and their mean saliency was calculated. For the natural-colour composite, the red, green, and blue bands were used as model inputs. The single available band was used for each high-pass-filtered product and spectral index, whereas the first three principal components were used for each PCA product. To enable comparison among rasters, the mean saliency was converted into an empirical percentile rank, defined as the proportion of valid raster pixels with a saliency value equal to or lower than the feature mean. Percentile ranks ranged from 0 to 1, with higher values indicating comparatively high saliency. Derived products were ranked by their mean percentile rank across the 14 crop marks, whereas the five negative controls were analysed separately. The highest-ranking products were then compared with those selected through expert visual interpretation.

SAM 3 outputs were assessed visually using the merged segmentation masks to avoid duplicate detections caused by overlapping tiles. Each segmented instance was compared with the reference dataset and classified as matching a field-recorded crop mark, a newly identified candidate, a negative-control feature, or an unrelated feature. The shape and spatial context of the detected patterns were also examined to identify common causes of false-positive responses.

Unless otherwise stated, all raster images were displayed using a linear contrast stretch from μ−3σ to μ+3σ. Consequently, image brightness represents relative variation within each product rather than directly comparable absolute values. Colour scales were therefore omitted.

## 3. Results

### 3.1. Toolbox Performance

Conventional preprocessing of the selected WV3 image was completed in under 15 min. This included atmospheric correction, cropping to the study area, and Bayesian pansharpening, which best preserved colour fidelity relative to the original natural-colour multispectral composite (Figure 5). By comparison, Z-PNN pansharpening required approximately 9 h when using 10 fine-tuning epochs.

Processing times varied among derived-product categories. Computing 21 spectral indices took approximately 10 min, or about 30 s per index. Generating 219 PCA products required around 6 h, with an average of 1.6 min per product. Applying the four high-pass filters to the atmospherically corrected panchromatic band took approximately 5 min.

**Figure 8:**
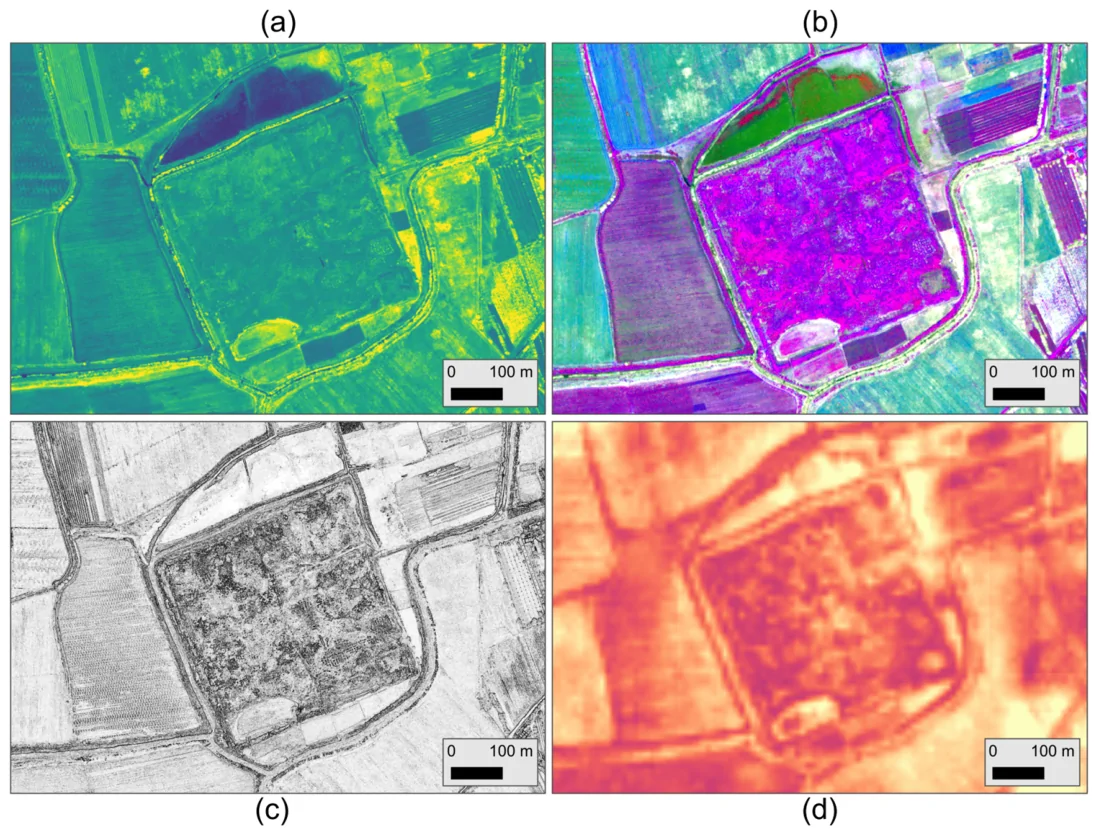
Representative examples of the derived image products: (**a**) the GDVI spectral index; (**b**) a false-colour RGB composite generated from principal components 1, 2, and 3; (**c**) Sobel high-pass filtering; and (**d**) the MAE saliency map computed from the PCA-derived image product shown in (**b**).

In total, 244 derived products were obtained, including PCA images, spectral indices, and high-pass filtered products (Figure 8). These datasets occupied 276.5 GB of disc space using high compression and a cloud-optimised format. PCA images accounted for approximately 270 GB, spectral indices for 6 GB, and high-pass filters for about 500 MB. On average, each PCA image occupied 1 GB, high-pass filters 0.5 GB, and spectral indices 200 MB. Compression reduced file size by approximately 10%, corresponding to a saving of around 27 GB.

The Masked Autoencoder (MAE) analysis was applied to all derived products to generate saliency statistics. This was the most computationally demanding step. Processing times averaged 4 min per PCA image, 3 min per high-pass filter, and 2 min per spectral index, resulting in a total runtime of approximately 5 h. The combined storage footprint of derived image products and MAE saliency maps reached 310 GB.

### 3.2. Identification of Candidate Crop Marks

**Figure 9:**
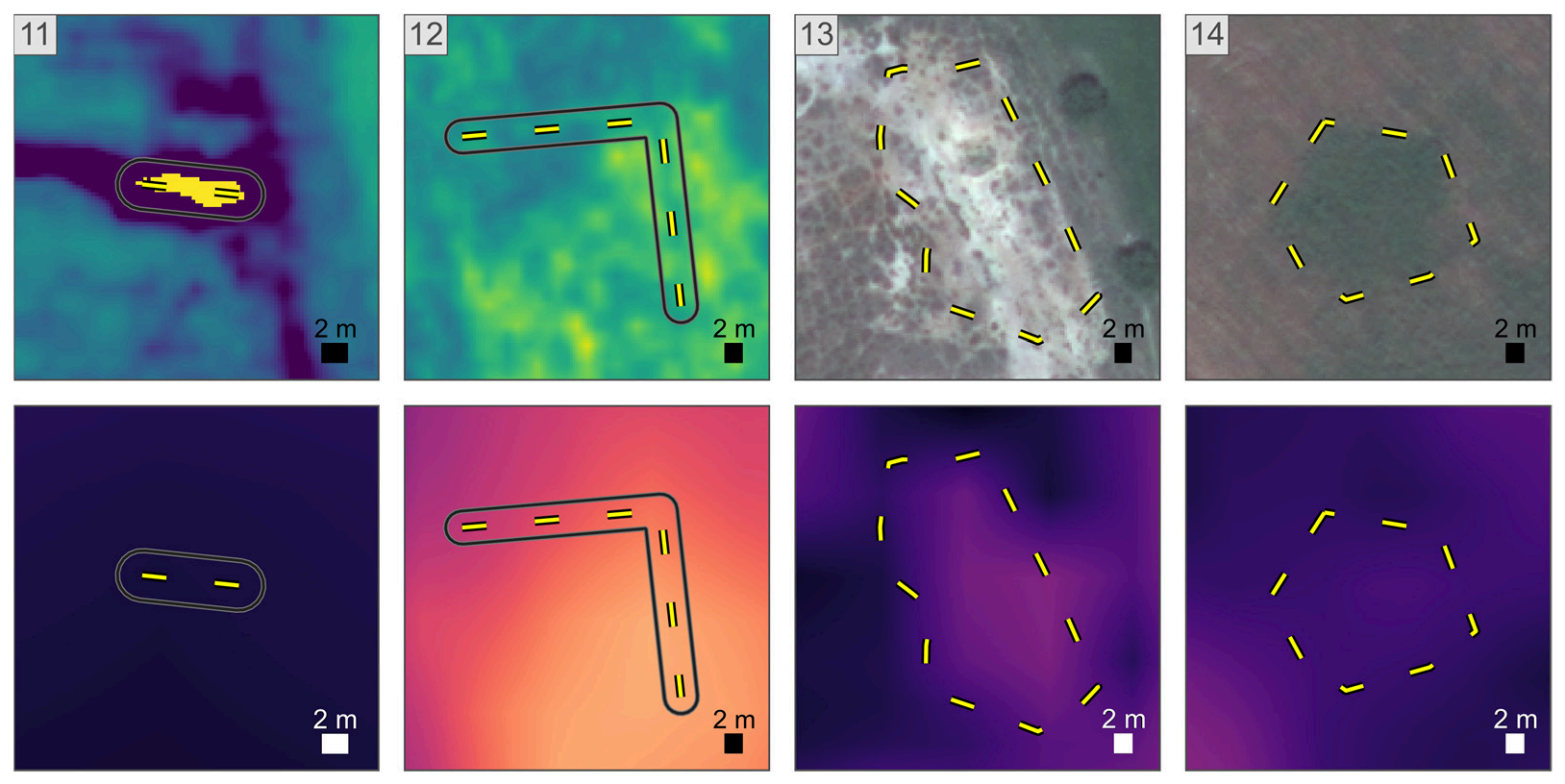
Locations of candidate crop marks identified using ArqPy products. The upper row shows the products used for their identification: TCARI/OSAVI for ID 11, MTVI for ID 12, both displayed using the viridis colour scale, and the Bayesian-pansharpened natural-colour composite for IDs 13 and 14. The lower row shows the corresponding MAE saliency maps displayed using the magma colour scale. Crop marks are outlined with yellow dashed lines; those outlined in black were not visible in the natural-colour composite and required derived products for their identification.

An expert visually examined all 244 derived products, resulting in the identification of four previously undocumented candidate crop marks. Two of these (IDs 13 and 14) were clearly visible in the Bayesian-pansharpened natural-colour composite (Figure 9). ID 13 may correspond to part of the site’s defensive wall, whereas ID 14 forms a distinctive pentagonal pattern within a cultivated field.

The other two candidates were identified using spectral-index products. ID 11 appeared as a small area of high TCARI/OSAVI values. No distinct crop-mark pattern was visible in the natural-colour composite, although the area showed relatively sparse vegetation within a cultivated field. Because TCARI/OSAVI is sensitive to chlorophyll content while reducing the effects of soil background and canopy structure [55], the high values may indicate reduced chlorophyll content or vegetation vigour. However, they could also be influenced by sparse vegetation or exposed soil; field validation is therefore required to determine the cause of this anomaly.

**Figure 10:**
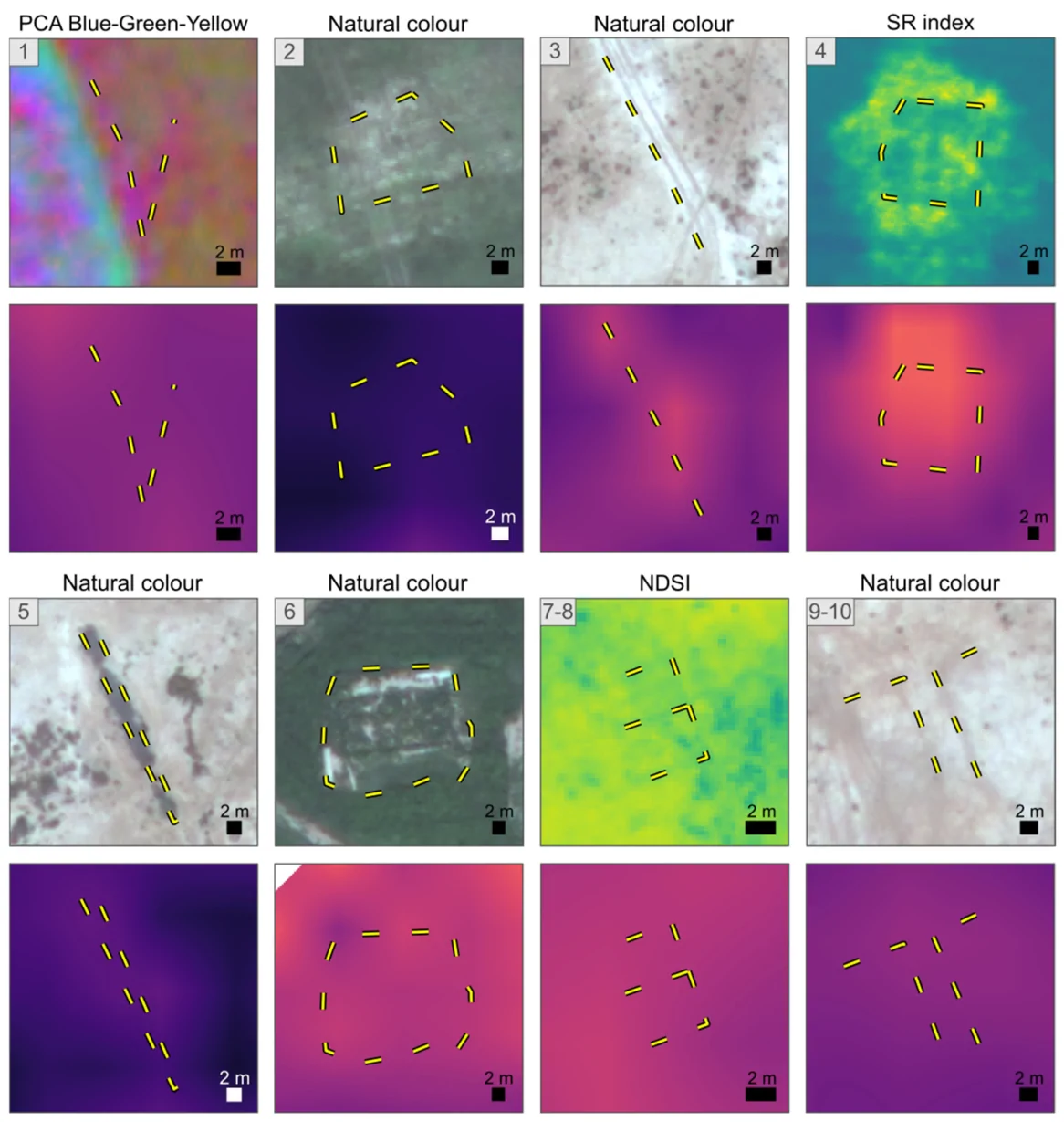
Ground-validated crop marks recorded during the 2024 and 2025 field campaigns, shown in the image product in which each was most clearly identified. The upper row presents the selected products, and the lower row shows their corresponding MAE saliency maps. The indices are displayed using the viridis colour scale. The PCA is shown as an RGB composite of the first three principal components, with the first, second, and third components assigned to the red, green, and blue channels, respectively. The MAE saliency maps are displayed using the magma colour scale. Crop marks are outlined with yellow dashed lines; those outlined in black were not visible in the natural-colour composite and required derived products for their identification.

Previously ground-validated crop marks were examined in the derived product in which each was most clearly visible (Figure 10). Six crop marks were identified in the pansharpened natural-colour composite. Four were located within the archaeological site: a possible main road (ID 3), two possible road intersections (IDs 9 and 10), and a straight feature covered by shrub vegetation (ID 5). The other two were rectangular features located within or near cultivated fields (IDs 2 and 6).

The remaining crop marks were not clearly visible in the natural-colour composite. ID 1 was identified in a PCA product generated from the blue, green, and yellow bands. It formed an irregular pattern adjacent to a field boundary south of Zar Tepe. ID 4 was located within a cultivated field in the western part of the study area, where low SR values formed a roughly square pattern surrounded by higher values. Finally, IDs 7 and 8 were identified using the NDSI product and may correspond to secondary roads within the main archaeological site.

### 3.3. Analysis of MAE Saliency Values

Overall, PCA-derived products exhibited the highest mean and maximum MAE saliency values (Table 2). Crop mark ID 1 was identified using a PCA product generated from the blue, green, and yellow bands (combination 22). This used the same bands as the highest-ranking PCA product (combination 94), except that the latter also included the NIR1 band.

The Bayesian-pansharpened natural-colour composite produced the second-highest saliency values and supported the identification of eight crop marks (Table 1). High-pass-filtered products and spectral indices showed lower and broadly similar values. No crop marks were identified using the high-pass-filtered products. In contrast, four spectral indices supported the identification of five crop marks: SR for ID 4, NDSI for IDs 7 and 8, TCARI/OSAVI for ID 11, and MTVI for ID 12. TCARI/OSAVI had the highest mean MAE saliency among the spectral-index products

When MAE saliency was compared between crop-mark and negative-control locations, the negative controls produced higher mean values and percentile ranks for all product groups except spectral indices (Table 3). This indicates that MAE responded strongly to non-archaeological geometric features, including the tractor and harvested-field boundary, sometimes more strongly than to crop marks. At the product-group level, the negative controls had the highest mean percentile rank in the Bayesian-pansharpened image, followed by the PCA products. In contrast, crop-mark locations had their highest mean percentile rank in the PCA group.

In Figure 9 and Figure 10, the MAE maps showed elevated saliency relative to the surrounding pixels for IDs 3, 4, 6, 7, 8, 9, 10, and 12, represented by brighter red-to-yellow tones on the magma colour scale. The remaining crop marks appeared in darker purple tones, although most were still brighter than their immediate surroundings. ID 11 was the main exception, showing no clear local saliency enhancement and appearing as a dark area.

Except for the Bayesian-pansharpened image, the products with the highest MAE rankings at crop-mark locations did not correspond to those selected through expert visual interpretation. Within the spectral-index group, BAI produced the highest mean percentile rank at crop-mark locations, whereas NDSI ranked highest at the negative controls. Because NDSI was also used to identify crop marks, this result highlights its potential to emphasise both archaeological and non-archaeological patterns.

### 3.4. Analysis of SAM 3 Instance Segmentation

**Figure 11:**
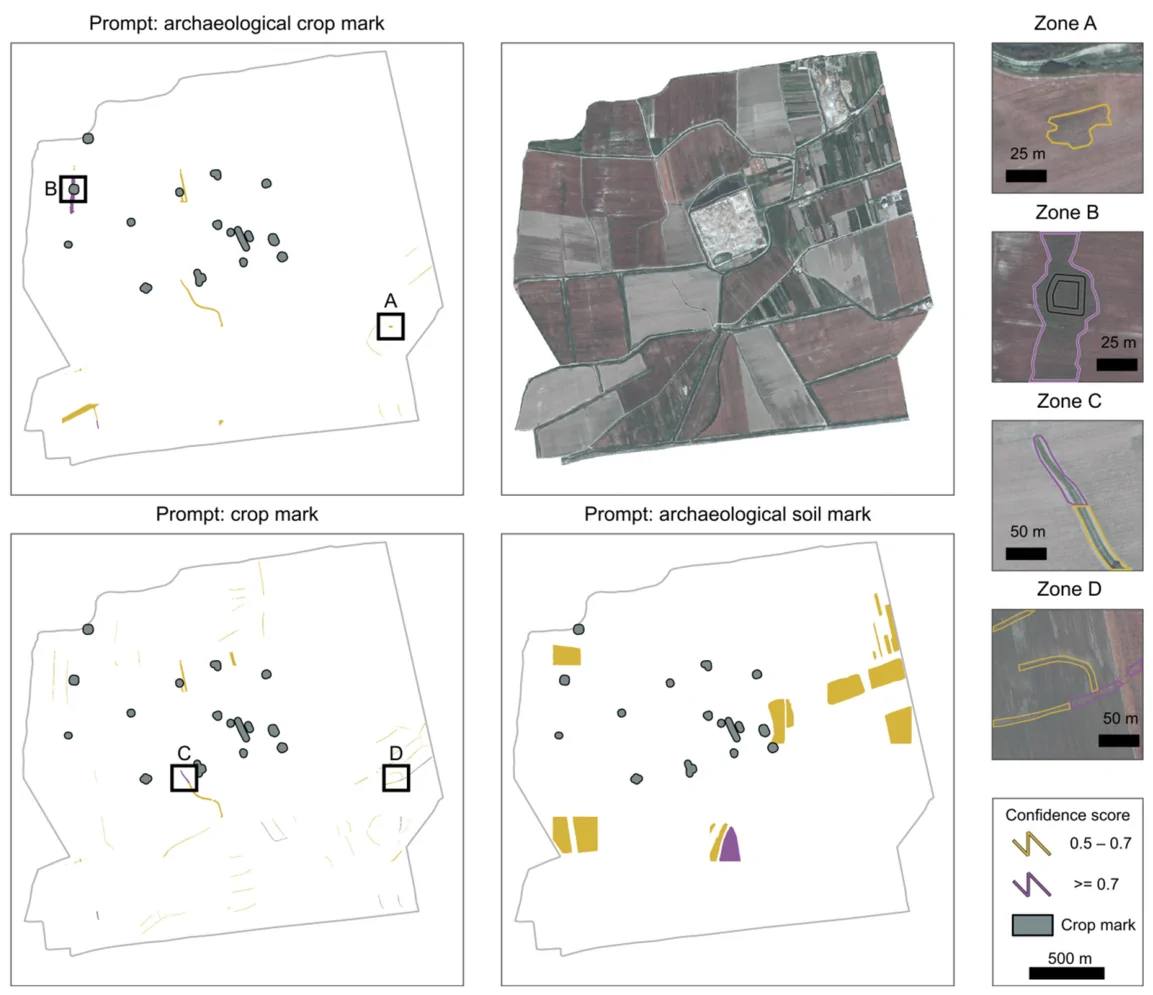
SAM 3 instances generated using the three text prompts, shown over the Bayesian-pansharpened image used as model input. Reference crop marks are displayed in grey, while predicted instances are classified by confidence score. Four areas are highlighted: zones A and B represent the two potentially relevant detections, whereas zones C and D illustrate responses to modern or historical roads, tracks, and field boundaries. Although the latter are crop-mark-like geometric features, they were not interpreted as archaeological crop marks.

The red, green, and blue bands of the Bayesian-pansharpened image were used as input to SAM 3. Predicted instances with confidence scores above 0.5 were retained. Three text prompts were tested to assess the model’s behaviour: *crop mark*, *archaeological crop mark*, and *archaeological soil mark* (Figure 11).

With the prompt *crop mark*, the predicted instances primarily corresponded to roads and tracks within the cultivated fields surrounding the site (Figure 11, zone C). The model also identified former paths and field boundaries (Figure 11, zone D) visible in historical CORONA imagery [42].

Adding *archaeological* to the prompt reduced the number of predicted instances. One instance overlapped crop mark ID 4 (Figure 11, zone B), although the predicted mask was larger than the digitised feature and enclosed the entire patch of vegetation within an otherwise bare field. The prompt also identified an area southeast of the site with an unusual vegetation pattern (Figure 11, zone A). This anomaly may reflect a subsurface feature, but field investigation is required to determine its origin.

Neither prompt identified crop marks within the main archaeological site. Therefore, the word *crop* was replaced with *soil* to form the third prompt, *archaeological soil mark*. However, this prompt primarily segmented large geometric areas within cultivated fields and did not identify any archaeologically relevant crop marks inside or outside the site.

## 4. Discussion

Very-high-resolution multispectral imagery acquired by the WV3 proved effective for enhancing subtle variations in vegetation and soil conditions potentially associated with buried archaeological remains. ArqPy supports reproducibility by making the processing sequence and its parameters explicit and traceable. It therefore contributes to broader efforts to standardise remote sensing preprocessing and analysis in archaeological research. By integrating established open source processing capabilities into an accessible Windows application, ArqPy reduces some of the technical barriers associated with image preprocessing and dataset preparation.

Existing applications support image preprocessing and the generation of derived products at both research and operational scales. However, their adoption is often constrained by fragmented workflows, usability limitations, and the expertise required to install, configure, and combine multiple software components [56]. Although recent initiatives have begun to address these limitations [57,58,59], the wider software ecosystem remains fragmented.

The current pipeline uses predefined parameters for certain operations, including the coefficients applied in weighted Brovey pansharpening. These settings reflect the requirements of the Zar Tepe case study and favour consistency and robustness. Future development will increase flexibility by allowing users to modify processing parameters without editing the source code. ArqPy currently uses four separate environments to manage the dependencies of its conventional and AI-based tools. Their consolidation will be investigated where compatibility permits, with the aim of reducing storage requirements and simplifying installation.

The complete execution of the toolbox produces a large number of derived products, requiring substantial storage space and making visual inspection more time-consuming. An efficient strategy is therefore needed to identify the most informative products for the detection of crop and soil marks. In this case study, expert visual inspection remained the most reliable method for recognising and delineating crop marks. However, its results depend on local vegetation, soil, acquisition conditions, and archaeological characteristics; consequently, the most informative products at Zar Tepe may not perform equally well elsewhere [31]. Forthcoming field campaigns at comparable sites in Uzbekistan will provide an opportunity to assess the transferability of these results.

MAE saliency highlighted several crop marks but produced weak responses for others. Agreement between the MAE rankings and the products selected through expert interpretation was limited, although TCARI/OSAVI was a notable exception. Moreover, MAE produced strong responses to non-archaeological geometric features, including a tractor and a harvested-field boundary. The saliency maps therefore cannot independently distinguish archaeological crop marks from other visually distinctive patterns. They should be treated as exploratory indicators and interpreted alongside the source products, archaeological expertise, and field-validation data.

SAM 3 provided more direct spatial candidates than MAE because it generated segmented instances rather than general saliency patterns. It identified one area overlapping a visually interpreted crop mark and another potentially relevant vegetation anomaly. However, it also segmented numerous modern and historical paths, tracks, and field boundaries. These results show that text-guided segmentation alone cannot reliably determine the archaeological origin of a feature. Future work should investigate task-specific training or classification using representative archaeological and non-archaeological examples. Few-shot image classification may be particularly useful where labelled samples are limited [60], while ongoing initiatives to develop crop-mark datasets could support more robust model adaptation [31].

The pansharpened natural-colour composite, PCA products, and spectral indices were the most useful sources for visual crop-mark identification in this case study. High-pass-filtered images did not reveal additional crop marks, although they clarified the linear and edge-based characteristics of some features already visible in the natural-colour composite. They should therefore not be excluded from future inspections, as their usefulness may vary among sites and feature types. The complementary information provided by PCA and spectral indices also demonstrates the limitations of applying AI models only to red, green, and blue inputs. Future models should be adapted to exploit red-edge, near-infrared, and other multispectral information.

Finally, this study was based on a relatively small case study, and further testing is required to evaluate the derived products and AI-based methods across the wider region. Additional field campaigns are planned to expand the validation dataset and improve the robustness of the workflow. Bidirectional reflectance distribution function and topographic corrections were not applied because the study area is flat and lacks substantial relief or tall vegetation. These operations could be incorporated into future versions of ArqPy to extend its applicability to landscapes with complex terrain and vegetation structure. Its object-oriented sensor architecture provides a suitable foundation for this extension, allowing new processing operations and sensor-specific parameters to be integrated without redesigning the entire workflow.

## 5. Conclusions

This study presents ArqPy, a Python-based toolbox designed to standardise and automate key preprocessing and enhancement operations for archaeological remote sensing. The workflow integrates atmospheric correction, conventional and AI-based pansharpening, spatial filtering, PCA and spectral index generation for very high-resolution satellite imagery. Its graphical interfaces and Windows-based distribution reduce technical barriers, while its object-oriented sensor architecture facilitates the future integration of additional sensors beyond WV3 and LEGION.

Application to Zar Tepe generated 244 derived products and supported the expert identification of four previously undocumented candidate crop marks before the forthcoming field campaign. The pansharpened natural-colour composite, PCA products, and spectral indices provided the most useful information for visual interpretation. However, the most informative product varied among crop marks, demonstrating the value of systematically examining complementary image products rather than selecting a single universal solution.

The integrated AI-based tools supported different stages of the workflow but also showed important limitations. MAE saliency highlighted several crop marks but responded strongly to non-archaeological geometric features and showed limited agreement with expert product selection. SAM 3 generated potentially relevant segmentation candidates but also detected roads, tracks, and field boundaries. These tools can therefore assist preliminary reconnaissance and product exploration, but they cannot replace expert interpretation and field validation.

Overall, ArqPy promotes reproducibility and transparency by making the processing chain explicit and repeatable. Future work will focus on validating the workflow at additional archaeological sites, incorporating more sensors and correction methods, increasing parameter flexibility, and adapting AI models to archaeological training data and multispectral information beyond the visible bands.

## Figures and Tables

**Table 1:** Description of the validation set of crop marks.

Group	Seen in RGB	Located Only in Derived Product	Total
2024 campaign	4	4	8
2025 campaign	2	0	2
ArqPy derived	2	2	4
False crop marks	-	-	5

**Table 2:** Mean and maximum MAE saliency values for each derived-product group, together with the product showing the highest mean saliency within each group. Statistics for the Bayesian-pansharpened natural-colour composite, analysed using its red, green, and blue bands, are included for comparison.

Group	Mean Saliency	Max Saliency	Best Derived Product
PCA	14.3	27.57	B-G-Y-N combination
High-pass filters	12.1	17.4	Laplacian filter
Spectral indices	12	17.5	TCARI-OSAVI
Bayesian PAN	12.8	17.9	-

**Table 3:** Mean MAE saliency values and empirical percentile ranks at the locations of 14 crop marks and five negative controls, summarised by derived-product group. For each group and feature class, the product with the highest mean percentile rank is reported. Results for the Bayesian-pansharpened natural-colour composite are included for comparison.

Group	Crop Marks	MAE Saliency	Percentile Rank	Best Product
PCA	Identified	14.4	0.54	G-Y-R-N2
	Negative	15.5	0.69	Y-N-N2
High-pass	Identified	11.7	0.38	Log5
	Negative	12.1	0.66	Log5
Indices	Identified	11.5	0.44	BAI
	Negative	12.28	0.38	NDSI
Bayesian PAN		12.3	0.43	-
		13.58	0.78	-

## Data Availability

The original data presented in the study are openly available in the ArqPy-Toolbox GitHub repository at https://github.com/CristianICS/ArqPy-Toolbox (accessed on 24 August 2026).

## Abbreviations

The following abbreviations are used in this manuscript:

MAE	Masked Autoencoders
WV3	WorldView-3
LEGION	WorldView Legion
